# Challenges in the Management of Hepatitis B Among Children and Adolescents: A 10-Year Single-Center Experience

**DOI:** 10.3390/microorganisms14091866

**Published:** 2026-08-22

**Authors:** Anna Dobrzeniecka, Ewa Talarek, Małgorzata Aniszewska, Barbara Kowalik-Mikołajewska, Agnieszka Ołdakowska, Beata Krynicka-Czech, Magdalena Marczyńska, Maria Pokorska-Śpiewak

**Affiliations:** 1 Department of Children’s Infectious Diseases, Medical University of Warsaw, 02-091 Warsaw, Poland; 2 Pediatric Infectious Diseases Department, Regional Hospital of Infectious Diseases in Warsaw, 01-201 Warsaw, Poland

**Keywords:** chronic hepatitis B (CHB), children, occult hepatitis B (OBI)

## Abstract

Following successful neonatal HBV vaccination and perinatal screening, pediatric hepatitis B has become uncommon in Central Europe. However, migration and complex clinical scenarios are reshaping its epidemiology. We aimed to characterize the clinical course, disease phases, and treatment eligibility of children with HBV infection. This retrospective single-center study included patients aged 0 to <18 years referred to a tertiary pediatric infectious diseases center in Warsaw, Poland, between January 2016 and June 2026. Demographic, clinical, and laboratory data were analyzed, and patients were classified according to the 2025 EASL guidelines. Sixteen children were included. New referrals tripled from 2016–2020 (*n* = 3) to 2021–2026 (*n* = 9), and 50% were refugees, predominantly from Ukraine. Vertical transmission accounted for 44% of infections, whereas 25% were iatrogenic. According to the 2025 EASL criteria, 38% (6/16) of patients were unclassifiable. Occult HBV infection was identified in 31%, while only 13% (2/16) received analogue therapy because of restrictive national treatment criteria. One adolescent developed liver fibrosis (7.6 kPa), likely related to metabolic steatohepatitis. Contemporary pediatric HBV management might require adaptation to changing epidemiology, recognition of the limitations of adult-derived disease phase classifications, and prevention of HBV reactivation during immunosuppression.

## 1. Introduction

Hepatitis B virus (HBV) infections still pose a significant global health problem, with more than 240 million people (2.9% of the global population) living with chronic hepatitis B (CHB) worldwide, contributing to more than 1 million deaths due to HBV-related complications [1]. According to a model study by Polaris Observatory Collaborators, only 36 million HBsAg-positive patients had been diagnosed, and only 6.8 million of the 83.3 million patients eligible for antiviral therapy were receiving treatment in 2022 [2]. The prevalence of CHB shows significant geographical differences, with sub-Saharan Africa, East Asia, and the Pacific Islands reporting the highest rates of HBV cases. In Europe, Eastern and Southeastern countries are considered the most affected, with an intermediate prevalence of HBV [2]. However, recent trends, including globalization, migration, and refugee movements, have shifted the prevalence of HBV in Europe [3].

In Poland, no more than 4000 new CHB cases are reported annually (Table 1), with the incidence exceeding 8 per 100,000 citizens in recent years [4]. Children and adolescents (aged 0–19 years) constituted 1.1% of acute hepatitis B cases and 0.5% of all reported CHB cases between 2015 and 2024 (Table 1). In addition, between 2012 and 2023, the average age of patients with CHB in Poland increased from 41 to 52 years [5].

The low prevalence of HBV infections among younger age groups may be a consequence of the national neonatal vaccination program, which was launched in Poland between 1994 and 1996. Moreover, in accordance with the Polish Standard of Perinatal Care, all pregnant women should be tested for HBsAg twice: by the 10th week of pregnancy (or at the time of the first medical consultation) and between the 33rd and 37th weeks of pregnancy. This enables the implementation of preventive strategies to reduce the risk of infection in the newborn [6]. Mother-to-child transmission (MTCT) and early horizontal HBV transmission are considered the main burdens of CHB worldwide, as chronic infection develops in 90% of infants but only in approximately 5% of adolescents and adults who acquire the virus [7]. Thus, to implement the World Health Organization’s (WHO’s) Global Health Sector Strategy (2022–2030) [8], which outlines clear targets to eliminate viral hepatitis as a public health problem, it is essential to concentrate on the pediatric population infected with HBV. In the recent Practice Guidelines on the Management of Hepatitis B Virus Infection, the European Association for the Study of the Liver (EASL) highlights critical gaps in the cascade of care for patients with CHB and emphasizes the urgent need for improvement in prevention efforts, early diagnosis, and access to treatment [9]. However, compared with adult populations, little attention has been given to the diagnosis and treatment of CHB among children and adolescents [7]. Therefore, considering these knowledge gaps, this study aimed to identify pediatric patients with HBV infection to analyze the clinical course of the disease and the application of antiviral treatment, ultimately optimizing pediatric-specific management practices.

## 2. Materials and Method

In this single-center retrospective study, we aimed to identify all patients who were referred at least once to the Department of Children’s Infectious Diseases, Medical University of Warsaw, Poland (both inpatient and outpatient settings), due to an HBV-related disease. Our department is located at the Regional Hospital of Infectious Diseases in Warsaw, Poland, and serves as a tertiary health care center for pediatric patients with infectious diseases. Specifically, we manage children with viral hepatitis and provide therapeutic programs for HBV and HCV in patients <18 years of age.

We retrospectively searched all available medical records from January 2016 to June 2026 to identify patients with ICD-10 B18.0 and B18.1 diagnoses (chronic viral hepatitis B with or without delta virus) as well as B16 (acute hepatitis B). For each included patient, epidemiological, clinical, and laboratory data were extracted and summarized. Specifically, patients were categorized according to the 2025 EASL phase classification of HBV infection. Additionally, the application and standard of antiviral treatment were analyzed. In general, follow-up visits were scheduled every 6 months. Elastography evaluation has been available since January 2021, and since then it was performed at every patient’s visit. The analysis focused on the results from each patient’s most recent visit to our department.

All patients aged 0 to <18 years were eligible for the study if they had any evidence of HBV infection or other HBV-related problems (e.g., organ transplant recipients from HBV-positive donors). Children and adolescents referred for HBV postexposure prophylaxis were excluded. Children in whom HBV infection was ruled out were also deemed ineligible for this analysis.

Children with CHB included in this analysis were categorized according to the 2025 EASL description of the HBV infection phases and the Polish Guidelines, based on HBeAg status and alanine aminotransferase (ALT) activity (Table 2) [9]. Additionally, we included phase 5—HBsAg-negative infection (occult hepatitis B infection, OBI)—which is defined as a nonhomogeneous phase characterized by a total anti-HBc-positive and HBsAg-negative status, with undetectable or periodically low HBV DNA concentrations in the serum and its presence in the liver [10]. In these cases, immunosuppression can lead to HBV reactivation.

The investigation was performed in accordance with the ethical standards of the 1964 Declaration of Helsinki and its later amendments. Written informed consent was waived because of the retrospective nature of this analysis. The local ethics committee of the Medical University of Warsaw approved this study (approval number: AKBE/171/2026, 18 May 2026).

Antiviral treatment was administered according to the current guidelines of the EASL, the European Society for Paediatric Gastroenterology Hepatology and Nutrition (ESPGHAN), and the Polish Group of Experts for HBV [9,10,11]. The Polish national therapeutic program for CHB is open to beneficiaries over 3 years of age who have serum HBV DNA and HBsAg detectable for more than 6 months and who meet the following criteria: an HBV DNA viral load above 2000 IU/mL and confirmation of active hepatitis (ALT activity exceeding the upper limit of normal), histological changes in the liver confirming the diagnosis of chronic hepatitis, or liver tissue stiffness (>7.0 kPa) on liver elastography. Patients with liver cirrhosis who are awaiting liver transplantation; who are receiving planned or initiated immunosuppressive therapy, including biological therapy or anticancer chemotherapy; or who are initiating planned treatment for HCV infection are eligible for treatment irrespective of the HBV DNA viral load.

Currently recommended antiviral drugs for children and adolescents include entecavir (ETV, for children aged 2 years and older), tenofovir disoproxil fumarate (TDF, for children aged 2 years and older), and tenofovir alafenamide (TAF, for children aged 6 years and older and weighing at least 25 kg). Pegylated interferon α-2a is approved for children aged 3 years and older but is currently not used in our practice.

Categorical data are presented as numbers (percentages of total). For continuous variables, medians (interquartile ranges, IQRs) were calculated using MedCalc Statistical Software version 23.2.8 (MedCalc, Ostend, Belgium).

## 3. Results

### 3.1. Study Group

We identified 18 participants eligible for the study. Two of them—a 1-year-old girl born to an HBV-infected mother and a 13-year-old girl treated for hepatitis C—were excluded from the analysis. Both likely had false-positive, low-titer HBsAg results unaccompanied by any other marker of HBV infection, as they had undetectable HBV viral loads and negative anti-HBc antibodies. These two patients are being followed up twice annually with persistently undetectable HBV DNA and normal ALT activity. The baseline characteristics of the final study group of 16 participants are presented in Table 3.

Four (25%) patients were under our care before 2016. Between 2016 and 2020, 3 (19%) new patients were referred, and 9 (56%) new patients were admitted between 2021 and 2026. Eight patients (50% of the total cohort) were refugees who had acquired the infection in their country of origin (Ukraine). Seven (44%) patients were infected vertically, including one who spontaneously eliminated the virus during the second year of life. Among them, 3 received both vaccination and anti-HBs immunoglobulin after birth, another 3 were only vaccinated against HBV, and the remaining one did not receive any prophylaxis. Four (25%) patients had an iatrogenic source of infection, including 3 (19%) who were infected through blood transfusions; all of these cases occurred in refugees in their country of origin prior to arriving in Poland. Among those 4 children only 1 had been immunized after birth, and in the remaining 3 cases, no prophylaxis against HBV had been administered.

Four patients (25%), including 3 refugees, had only one visit to our department, whereas 12 (75%) were observed for a median duration of 17.5 (IQR 8; 37.8) months. During this period, spontaneous seroconversion of HBeAg was observed in 1 patient, and elimination of HBV infection occurred in 2 patients. Eight patients (50%) concluded their management at our department; among them, five were refugees who relocated to other countries, and three were patients transferred to adult care.

### 3.2. Phases of HBV Infection

**Figure 1:**
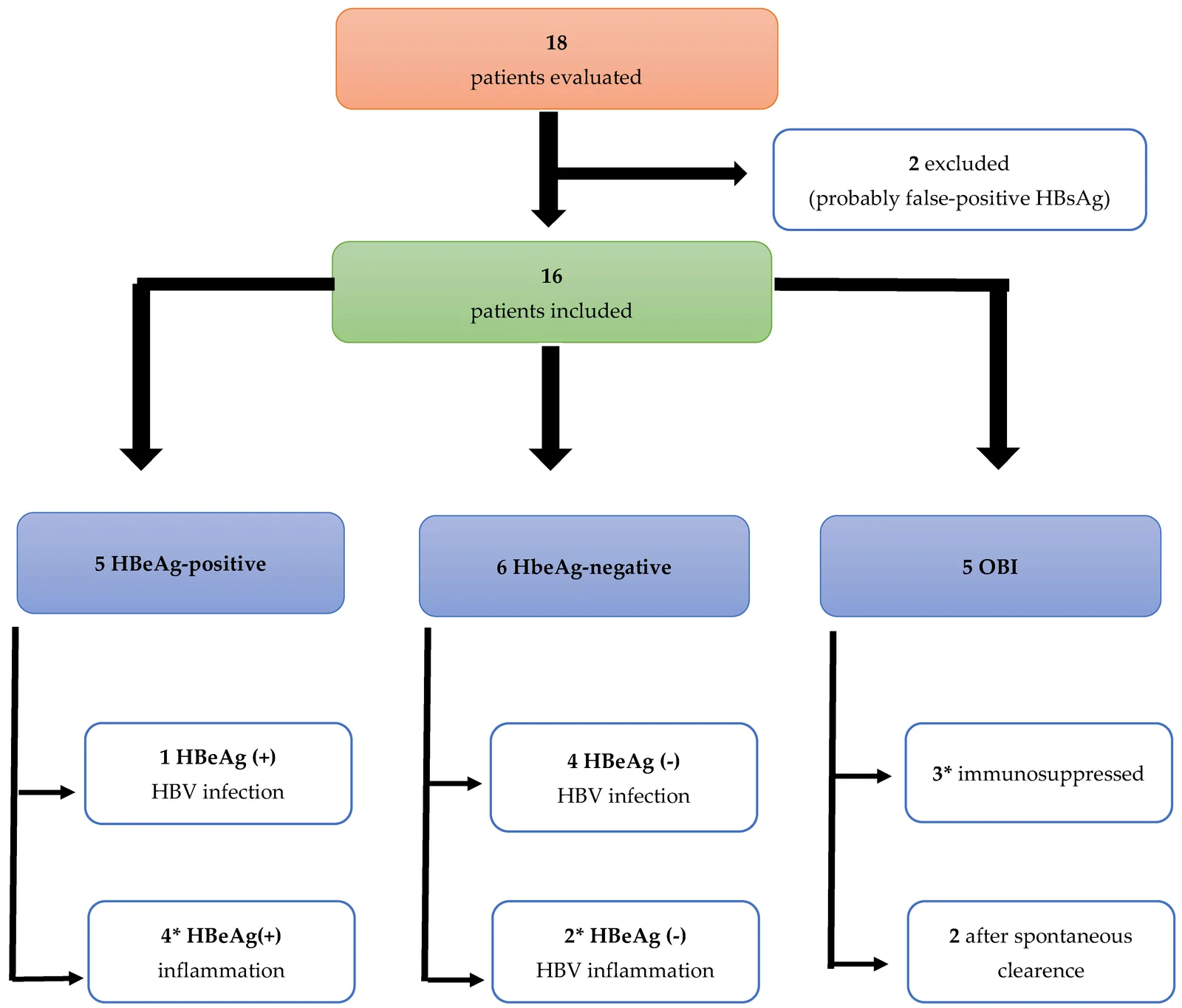
Patient selection and classification in our study group according to HBV infection phase. * Each asterisk represents one patient who received treatment with nucleos(t)ide analogs. OBI—occult HBV infection.

The patient selection process and classification according to HBV infection phase are presented in Figure 1. Only five participants (31%) were HBeAg positive: one with HBeAg-positive infection and four with HBeAg-positive hepatitis (inflammation). Notably, 3 of these patients did not meet the exact definition of the phase because the HBV viral load exceeded 10^7^ IU/mL and the transient elastography results were normal, indicating the absence of liver disease. Six other patients (38%) presented with HBeAg-negative infection (four participants) or HBeAg-negative hepatitis (two participants). Among them, 3 participants did not fulfill the phase criteria: 1 patient with HBeAg-negative infection had evidence of liver disease progression (F2 on the Metavir scale), and both patients with HBeAg-negative hepatitis presented with lower-than-expected HBV viral loads and without significant liver disease progression. Five patients (31%) were classified as having OBI: one who was coinfected with HIV and HCV, two who were immunosuppressed following organ transplantation, and two in whom spontaneous HBV elimination had been confirmed (one 2-year-old child of an HBV-infected mother who had presented with detectable HBV DNA in infancy and one 17-year-old patient who was previously diagnosed with acute hepatitis B). In Table 4, all the patients are presented according to the phase of HBV infection at their last visit.

### 3.3. Liver Disease Progression

Liver stiffness measurement (LSM) was performed in 11 (69%) patients using transient elastography (FibroScan 530 device, Echosens, Paris, France) (Table 4). At the last visit, 10 participants had LSM values of less than 7 kPa, indicating the absence of fibrosis (corresponding to F0/1 on the Metavir scale). In one 15-year-old boy, the LSM was 7.6 kPa, corresponding to an F2 score on the Metavir scale. The patient presented with HBeAg-negative HBV infection and undetectable HBV DNA; thus, he did not qualify for antiviral treatment. The elevated LSM was probably associated with metabolic factors (metabolic dysfunction-associated steatohepatitis, MASH), as the patient was obese (body mass index [BMI], 27.9 kg/m^2^; z-score, 1.94), and his liver steatosis stage 2 was confirmed by a transient elastography examination (controlled attenuation parameter [CAP], 280 dB/m).

### 3.4. Treatment

Three (19%) patients had a history of previous ineffective treatment, including one with IFN-based therapy, one with an analog, and one with both ineffective IFN therapy and analog treatment involving lamivudine (LAM) and entecavir (ETV, used in the country of origin). In this patient, we confirmed drug resistance to both LAM and ETV (due to the following mutations: 180 M, 202 G, and 204 V). This patient was eligible for treatment with tenofovir. Another patient was effectively treated with ETV (his HBV DNA was undetectable, but seroconversion of HBsAg was not achieved). Thus, currently, no child is receiving IFN therapy, whereas 2 (13%) patients are being treated with analogs. Additionally, one 8-year-old girl was receiving prophylaxis with ETV after undergoing a liver transplantation for hepatocellular carcinoma, having received the organ from an HBsAg-negative/anti-HBc-positive donor. Another anti-HBc-positive kidney transplant recipient is being regularly monitored and is not receiving anti-HBV prophylactic treatment due to a low risk of reactivation.

## 4. Discussion

Our analysis confirms that while the absolute number of pediatric patients with HBV infection in Central Europe has significantly declined—largely owing to successful national neonatal vaccination programs and robust perinatal screening—the clinical landscape has shifted. The number of new admissions in our center tripled during the 2021–2026 period compared with 2016–2020. This trend reveals critical contemporary challenges in managing HBV in children and adolescents, driven by migratory flows, diagnostic ambiguities, complex clinical scenarios including OBI, and strict therapeutic criteria.

A key finding of our study is the profound impact of global migration and refugee movements on local HBV epidemiology. Refugees accounted for 50% of our final study cohort, with all of them acquiring the infection in their country of origin (Ukraine). The increased number of migrants, particularly from Ukraine, contributed to the increase in the incidence and diagnosis rate of hepatitis B cases in Poland in 2023 and 2024 [12]. While mother-to-child transmission (MTCT) remains the predominant global driver of chronic hepatitis B [1,7], a notable 25% of our patients had an iatrogenic source of infection. Most remarkably, 19% were infected via blood transfusions prior to arriving in Poland. This underlines the ongoing disparities in health care safety standards globally and highlights the need for immediate, comprehensive infectious disease screening for all migrant children entering low-prevalence European countries. Notably, four of the seven patients who were infected vertically and three of the four patients with iatrogenic sources of infection did not receive appropriate prophylaxis against HBV infection during the neonatal period. This may reflect the problem of a decline in hepatitis B vaccination in Poland; in 2023 and 2024, further decreases were noted, with coverage among 1-year-olds being less than 90% (89.5% in 2023 and 87.3% in 2024) [12]. In addition, even more significant gaps in hepatitis B immunity are observed among Ukrainian pediatric refugees residing in Poland. A study by Stopyra et al. of 1322 migrants revealed that only 83.2% reported being vaccinated against hepatitis B, and 5 of them (0.4%) were diagnosed with CHB, four of whom were unvaccinated [13].

Another frequent challenge in clinical practice is categorizing pediatric patients into natural history phases. Standard frameworks, such as the 2025 EASL guidelines, are primarily derived from adult data and often fail to cleanly accommodate pediatric presentations. In our study, six of 16 patients (38%), including 3 HBeAg-positive and 3 HBeAg-negative participants, did not fully meet the strict 2025 EASL phase definitions. Similar observations were recently made in an Italian cohort of 248 pediatric patients, in which 45% were found to be unclassifiable by the EASL criteria [14]. Natural host–pathogen interactions and immune system development in children may contribute to these discrepancies. This is due to the fact that functionally distinct immune systems in early life may influence the natural progression of the disease related to HBV infection and thus, the timing of serological milestones [15]. Future studies that adapt classification systems for pediatric patients are needed to improve disease staging and guide management decisions [14]. Interestingly, the highly replicative HBeAg-positive infection phase, which may persist for years without causing immediate structural liver damage, was diagnosed in only one of our patients. Similarly, atypical presentations were noted in the HBeAg-negative cohort. A 15-year-old boy classified with HBeAg-negative infection had an elevated LSM of 7.6 kPa (F2 fibrosis), which traditionally triggers antiviral therapy. However, his HBV DNA was undetectable, and subsequent analysis attributed the fibrosis to MASH driven by obesity (BMI 27.9 kg/m^2^) and significant liver steatosis (CAP 280 dB/m). This case highlights the growing need to differentiate between HBV-induced liver injury and concurrent metabolic comorbidities in adolescents before initiating lifelong antiviral therapy. In a histopathological study analyzing liver steatosis in children with CHB, steatosis was observed in 4/30 (13%) of patients, and moderate-to-severe steatosis in 1/30 (3%) [16]. In these patients, the presence of steatosis was positively associated with the BMI z-score [OR = 3.3; 95% CI, 1.02–10.64] and with no viral factors, including HBeAg status and HBV viral load. This suggests that hepatic steatosis in patients with CHB is mainly associated with the host metabolic factors [16]. Wang et al. who analyzed the effect of CHB on hepatic steatosis and liver injury in children with MASH concluded, based on histopathological evaluation, that CHB was inversely associated with the grade of steatosis and positively associated with hepatic necroinflammation, but without significant influence of hepatic fibrosis [17]. This may further confirm that in our patient, the liver fibrosis was associated with metabolic and non-viral factors.

A striking observation in our 10-year experience is the low proportion of pediatric patients actively receiving or qualifying for antiviral therapy. Among the final cohort of 16 children, only 2 (13%) are currently treated with nucleos(t)ide analogs. This low treatment rate is a consequence of the rigorous inclusion criteria maintained by the Polish national therapeutic program, which requires a combination of a high viral load (>2000 IU/mL) and active liver inflammation or significant fibrosis (>7.0 kPa). In addition, we also treated two other patients (refugees) in the HBeAg-positive hepatitis (inflammation) phase, but they were lost to follow-up. Among our HBeAg-negative patients, one received treatment with entecavir and was nonviremic, whereas the other did not qualify for treatment because of a very low or undetectable HBV viral load. This watchful waiting approach requires prolonged, strict medical surveillance in these cases. In the Italian cohort, 14 (6%) children received antiviral therapy, and 50% achieved HBeAg seroconversion [14]. Thus, similar to our study group, most Italian children were not treated, but a significant proportion achieved seroconversion. In our cohort, two patients spontaneously eliminated HBV (one teenager after acute hepatitis B and one child infected vertically in the second year of life), and another three patients achieved HBeAg seroconversion (two on treatment with analogs and one spontaneously). The decision to initiate antiviral treatment in children with CHB must be carefully balanced, considering the potential benefits and risks, including the often benign disease course and uncertain duration of treatment [14]. Novel guidelines for HBV treatment suggest a shift toward a more proactive treatment in pediatric patients [9,10,18]. According to the 2024 WHO guidelines, treatment is recommended for all adolescents with persistently elevated ALT and detectable HBV DNA [18]. A similar approach was recently proposed by the Polish Group of Experts for HBV, who recommend therapy in children aged 2–18 years when HBV DNA is detectable and ALT activity is elevated [10]. According to these guidelines, 5/16 (31%) of our total cohort, and 5/11 (45%) of children excluding OBI cases, would fulfill the indications for treatment. This is also in agreement with the 2025 EASL guidelines, which state that all individuals with detectable HBV DNA are candidates for antiviral therapy [9]. Moreover, according to the 2025 EASL guidelines [9], young individuals (<30 years) with HBeAg-positive HBV infection with persistent normal ALT activity may also benefit from early treatment, with reduction in HBV DNA integration and clonal expansion. In these cases, a shared decision-making approach should be implemented. Considering this approach, even 7 patients in our group might be considered for early treatment.

Another important issue associated with HBV infection is the management of OBI and the prevention of HBV reactivation. The risk of HBV reactivation occurs when a reduction in immune control occurs due to the altered balance between the virus and the host’s immune system [19]. OBI, characterized by negative HBsAg but detectable anti-HBc and low or undetectable serum HBV DNA levels, constituted 31% of our study cohort. Managing OBI in pediatric patients is highly critical because of the lifelong risk of HBV reactivation under conditions of immunosuppression [9,10,15]. In our series, the strategy for preventing HBV reactivation was tailored to individual risk. An 8-year-old girl who underwent liver transplantation from an anti-HBc-positive donor received pharmacological prophylaxis with ETV, as this scenario carried a risk of reactivation. Conversely, an anti-HBc-positive kidney transplant recipient is being managed via regular, strict molecular and serological monitoring without primary antiviral prophylaxis, given the lower risk profile. No reactivation events occurred during the follow-up period, demonstrating that a nuanced, risk-stratified approach to immunosuppressed children can safely minimize unnecessary drug exposure while preventing severe clinical flare-ups [9,10].

This study is primarily limited by its retrospective, single-center design and small sample size, reflecting the overall low prevalence of pediatric HBV in Poland. Consequently, these findings may not fully represent larger regional trends. The relatively short median follow-up may limit conclusions regarding fibrosis progression, seroconversion, long-term treatment outcomes, and HBV reactivation. In addition, a significant proportion of participants were lost to follow-up, reflecting one of the challenges associated with the current management of this group of patients.

## 5. Conclusions

In conclusion, while domestic pediatric HBV cases continue to dwindle, the management of hepatitis B in children and adolescents has become increasingly nuanced. Clinicians must adapt to an epidemiological reality shaped by migration, recognize that adult phase definitions do not always align with pediatric biology, acknowledge the limitations of current treatment eligibility criteria, carefully implement tailored strategies to assess the risk of reactivation, and implement tailored preventive strategies in an expanding population of immunosuppressed children. Further studies are needed in this field on larger cohorts of pediatric patients with HBV in order to confirm these findings.

## Figures and Tables

**Table 1:** New cases of hepatitis B reported in Poland between 2015 and 2024 [4].

Diagnosis	Acute Hepatitis B		Chronic Hepatitis B					
Age group	children (0–19 years)	all (children and adults)	0–4 years	5–9 years	10–14 years	15–19 years	children (0–19 years)	All (children and adults)
2015	3	55	2	2	5	31	40	3453
2016	0	50	3	2	1	12	18	3756
2017	1	56	1	1	1	8	11	3307
2018	0	40	3	0	1	7	11	3156
2019	0	45	5	1	1	5	12	2809
2020	0	14	0	0	0	0	0	977
2021	0	10	3	0	0	1	4	1537
2022	0	29	2	3	1	9	15	2471
2023	0	36	2	3	2	7	14	3106
2024	0	32	1	3	1	7	12	3513
**2015–2024**	**4**	**367**	**22**	**15**	**13**	**87**	**137**	**28,085**

Values in bold represent the total numbers of reported hepatitis B cases for the period 2015-2024.

**Table 2:** Phases of chronic hepatitis B according to EASL and the Polish Group of Experts for HBV Guidelines [9,10].

Phase	1	2	3	4	5
	HBeAg-Positive		HBeAg-Negative		HBsAg-Negative Infection
	Infection	Hepatitis	Infection	Hepatitis	
HBsAg	High	High/Intermediate	Low (<1000 IU/mL)	Intermediate	negative
ALT	Normal	Elevated	Normal	Elevated	Normal
HBV DNA	>10^7^ IU/mL	10^4^–10^7^ IU/mL	<2000 IU/mL	>2000 IU/mL	Undetectable/low
Liver disease progression	none/ minimal	moderate/ severe	none	moderate/ severe	none
Progression to cirrhosis	none	possible	none	More rapid than in other phases	none
Treatment	Not generally indicated	May be indicated	Not indicated	May be indicated	Not indicated

**Table 3:** Characteristics of patients managed due to HBV infection between 2016 and mid 2026 (*n* = 16).

Characteristic	Value
Sex (Male/Female)	11 (69%)/5 (31%)
Age at the last visit—median of years (IQR)	9.5 (8; 14)
Refugees	8 (50%)
Immunosuppressed	2 (13%)
Post transplantation	3 (19%)
	(liver, kidney, marrow)
Source of HBV infection	
-mother-to-child transmission	7 (44%)
-iatrogenic/including blood transfusion	4 (25%)/3 (19%)
-unknown	5 (31%)
Coinfection	
-HCV	1 (6%)
-HIV	1 (6%)
Previous anti-HBV treatment	3 * (19%)
-IFN	2 (13%)
-NA	2 (13%)
Currently treated (or at the last visit)	2 (13%)
-ETV	1 (6%)
-TDF	1 (6%)
Prophylaxis of HBV reactivation with NA	1 (6%) (ETV)
ALT (IU/L)—median (IQR)	37 (20; 81)
>40 IU/L	6 (38%)
AST (IU/L)—median (IQR)	34 (29; 66)
>40 IU/L	6 (38%)
HBV DNA PCR (positive)	9 (56%)
≥2000 IU/mL	5 (31%)
<2000 IU/mL	4 (25%)
LSM	
≥7 kPa	1 (6%)
<7 kPa	10 (63%)

* 1 Patient previously treated with IFN, LAM and ETV. Laboratory and elastography data are presented for the last patients’ visits.

**Table 4:** Patient characteristics at their last visit according to the phase of HBV infection.

No.	Sex/Age (Years)	ALT	HBsAg (IU/mL)	Anti-HBs	Anti-HBc Total	HBeAg	Anti-HBe	HBV DNA (IU/mL)	LSM (kPa)	Phase *	Additional Information
1	F/9	N	+	−	+	+	−	2.27 × 10^8^	5.5	1	Refugee, LTFU
2	F/1	↑	+	−	+	+	−	2.97 × 10^8^	5.8	2 **	Child of a mother infected with HBV; received both vaccination and HBIG after birth
3	M/7	↑	+	−	NA	+	−	3.92 × 10^7^	NA	2	After ineffective IFN treatment, LTFU
4	M/10	↑	+ (13,000)	−	NA	+	−	3.08 × 10^8^	4.7	2 **	Qualified for NA treatment, refugee LTFU
5	M/13	↑	+ (28,000)	−	NA	+	−	4.93 × 10^8^	4.2	2 **	Qualified for TDF treatment (resistant to ETV and LAM; after ineffective IFN treatment)
6	M/9	N	+	−	+	−	+	4.5 × 10^2^	NA	3	No indications for treatment
7	F/12	N	+	−	+	−	+	0	5.9	3	After effective treatment
8	M/13	N	+ (3600)	−	+	−	+	<10	4.8	3	No indications for treatment
9	M/15	N	+ (24)	−	−	−	+	0	7.6	3 **	No indications for treatment
10	M/8	↑	+ (1300)	−	NA	−	+	0	6.1	4 **	Treated with ETV
11	M/16	↑	+ (1800)	−	+	−	+	1.28 × 10^2^	6.1	4 **	No indications for treatment
12	M/7	N	−	+	+	−	−	0	4.8	5	Treated with ARV due to HIV infection, and with DAA due to HCV infection
13	F/8	N	−	+	−	−	−	<20	NA	5	Prophylaxis with ETV; post-liver transplantation from donor HBsAg(−)/anti-HBc(+)
14	F/16	N	−	+	+	−	+	0	4.9	5	Immunosuppressed (post-kidney transplantation)
15	M/2	N	−	+	+	−	−	0	NA	5	Eliminated HBV (spontaneously)
16	M/17	N	−	+	+	−	−	0	NA	5	Post-acute hepatitis B with subsequent HBV elimination

* Phases of HBV infection/reasons for management of the child: 1—HBeAg (+) HBV infection; 2—HBeAg(+) inflammation; 3—HBeAg(−) HBV infection; 4—HBeAg(−) inflammation; 5—occult HBV infection. ** Patients who do not meet the exact criteria for the HBV infection phase according to the EASL (patients No. 2, 4, and 5 have an HBV viral load exceeding 107 IU/mL and normal values on transient elastography corresponding to no liver disease; patient No. 9 has significant liver fibrosis corresponding to F2 Metavir; and patients No. 10 and 11 have lower-than-expected HBV viral loads and no significant liver disease progression). Rows shaded in gray represent patients with elevated alanine aminotransferase (ALT) activity at their last visit. N—normal (<40 IU/L); NA—not applicable; ↑—elevated; +—positive; −—negative.

## Data Availability

The data presented in this study are available on request from the corresponding author. The data are not publicly available due to privacy restrictions.

## Abbreviations

The following abbreviations are used in this manuscript:

HBV	hepatitis B virus
CHB	chronic hepatitis B
HBsAg	hepatitis B surface antigen
HCV	hepatitis C virus
MTCT	Mother-to-child transmission
EASL	the European Association for the Study of the Liver
ESPGHAN	European Society for Paediatric Gastroenterology Hepatology and Nutrition
ALT	alanine aminotransferase
ETV	entecavir
IFN	interferon
TDF	tenofovir disoproxil fumarate
TAF	tenofovir alafenamide
LAM	lamivudine
LSM	liver stiffness measurement
LTFU	lost to follow up
OBI	occult hepatitis B infection
RT-PCR	real-time polymerase chain reaction
TE	transient elastography
WHO	World Health Organization
